# Allergic Inflammation Caused by Dimerized Translationally Controlled Tumor Protein is Attenuated by Cardamonin

**DOI:** 10.3389/fphar.2021.765521

**Published:** 2021-10-06

**Authors:** Haejun Pyun, Joo-Won Nam, Hyunsoo Cho, Jiyoung Park, Eun Kyoung Seo, Kyunglim Lee

**Affiliations:** ^1^ Graduate School of Pharmaceutical Sciences, College of Pharmacy, Ewha Womans University, Seoul, South Korea; ^2^ College of Pharmacy, Yeungnam University, Gyeongsan, South Korea; ^3^ Fluorescence Core Imaging Center, Department of Life Science, Ewha Womans University, Seoul, South Korea

**Keywords:** allergic airway inflammation, BEAS-2B cells, cardamonin, dimerized TCTP, histamine releasing factor

## Abstract

We demonstrated in our previous reports that dimeric form of translationally controlled tumor protein (dTCTP) initiates a variety of allergic phenomena. In the present study, we examined whether and how dTCTP’s role in allergic inflammation can be modulated or negated. The possible potential of cardamonin as an anti-allergic agent was assessed by ELISA using BEAS-2B cells and OVA-challenged allergic mouse model. The interaction between cardamonin and dTCTP was confirmed by SPR assay. Cardamonin was found to reduce the secretion of IL-8 caused by dTCTP in BEAS-2B cells by interacting with dTCTP. This interaction between dTCTP and cardamonin was confirmed through kinetic analysis (K_D_ = 4.72 ± 0.07 μM). Also, cardamonin reduced the migration of various inflammatory cells in the bronchoalveolar lavage fluid (BALF), inhibited OVA specific IgE secretion and bronchial remodeling. In addition, cardamonin was observed to have an anti-allergic response by inhibiting the activity of NF-κB. Cardamonin exerts anti-allergic anti-inflammatory effect by inhibiting dTCTP, suggesting that it may be useful in the therapy of allergic diseases.

## Introduction

Translationally controlled tumor protein (TCTP), also variously called histamine releasing factor (HRF), p21, p23, and fortilin, is involved in a variety of biological and pathological processes (Bommer and Thiele, 2004), the extracellular histamine release (MacDonald et al., 1995) and inducing the production of interleukins from basophils, mast cells, and eosinophils (MacDonald et al., 1995; Kawakami, 2014). TCTP acts in the extracellular space to form dimers with cytokine-like activity (Kim et al., 2009). Dimerized TCTP (dTCTP) is found in the body fluids of patients with allergic diseases (Kim et al., 2009; Ulambayar et al., 2019), mice with atopic dermatitis (Jin et al., 2017), and bronchial alveolar lavage fluid (BALF) of mice with allergic airway inflammation (Kim et al., 2009; Pyun et al., 2018). It has been known to play an important role in late phase allergic reactions (Kim et al., 2009). Based on these findings, we hypothesized that by regulating dTCTP, it is possible to suppress late phase allergic responses. We tested our hypothesis in BALB/c mouse models and screened natural product libraries and selected chemical compounds for their ability to inhibit dTCTP’s role in allergic reactions. Using this approach, we identified cardamonin, a chalcone as one with this ability.

Chalcones are chemicals in the family of flavonoids that are present in many plants, vegetables, fruits and teas (Sikander et al., 2011). Among chalcones, cardamonin, the cardamom spice, is present in many plants and has been extensively studied (Goncalves et al., 2014), especially in the anticancer field related to the mTOR pathway (Break et al., 2018; Niu et al., 2015; Shi et al., 2018; Xue et al., 2016; X.; Zhou et al., 2019), inflammatory bowel disease (Ren et al., 2015; Ali et al., 2017; Wang et al., 2018), rheumatoid arthritis (Li et al., 2015; Voon et al., 2017), Sjogren’s syndrome (Benchabane et al., 2018), and parasitic infection (de Castro et al., 2015). Its antitumor and anti-inflammatory effects are shown to occur by suppressing nitric oxide, TNFα and NF-kB (Li et al., 2015; Ren et al., 2015; Ali et al., 2017; Voon et al., 2017; Benchabane et al., 2018). In addition, cardamonin exhibits anti-inflammatory effects rheumatoid arthritis by inhibiting the activity of NF-kB and AhR/Nrf2/NQO1 pathways (Ren et al., 2015).

However, little attention has been paid to its possible role in allergic airway inflammatory responses associated with dTCTP. We wondered if and how cardamonin can modulate dTCTP and whether it has the potential to be useful in the treatment of allergic diseases by targeting dTCTP. We employed BEAS-2B cells and a mouse model of OVA-induced allergic airway inflammation to examine whether and how dTCTP’s role in allergic inflammation can be modulated.

## Materials and Methods

### Materials

Recombinant dTCTP protein prepared as previously described (Kim et al., 2009), was used to screen for substances effective in the allergic airway inflammation *in vitro*. Cardamonin was purchased from Sigma Aldrich (St. Louis, MO, United States) and helichrysetin was purchased from ChemFaces (Hubei, China). Alum and ovalbumin (OVA) were purchased from Thermofischer scientific (Waltham, MA, United States). IL-4, IL-8, and OVA-specific IgE ELISA kit was purchased from Biolegend (San Diego, CA, United States). IL-13 ELISA kit was purchased from R&D systems (Minneapolis, MN, United States).

### BEAS-2B Cell Culture

Human bronchial epithelial cells, BEAS-2B, were purchased from the American Type Culture Collection (ATCC, CRL-9609) and cultured in bronchial epithelial cell growth medium (BEGM, Lonza) at 37°C, 5% CO_2_, and a humidified atmosphere.

### Immunofluorescence Analysis

Cells were cultured on a 12-well plate containing cover slips (18 mm diameter) coated with poly-L-lysine. BEAS-2B cells were seeded at 12,500 cells per well. After 24 h, cells were serum starved for 2 h and pre-treated with cardamonin at 2 μg/ml for 30 min, and stimulated with dTCTP (10 μg/ml) for 45 min followed by fixed for 10 min with 4% formaldehyde in phosphate-buffered saline (PBS). Fixed cells were exposed for 30 min at room temperature to PBS containing 0.1% Triton X-100 and 5% horse serum (Gibco-BRL, Thermo Fisher Scientific), and incubated for overnight at 4°C with NF-kB antibody in the same solution, were washed three times with PBS, and were incubated for 30 min at room temperature with Alexa Fluor 488–conjugated secondary antibody (Molecular Probes, Thermo Fisher Scientific). Cells were then stained with 4′,6-diamidino-2-phenylindole (0.2 μg/ml) to stain nuclei. Images were captured with a Zeiss LSM 880 confocal microscope at Ewha Fluorescence Core Imaging Center.

### Cell Viability Assay

BEAS-2B cells (1 × 10^4^ cells/well) were incubated in 96-well plates with various concentrations of cardamonin, for 24 h. Cell viability was measured using a Cell Counting Kit-8 (CCK) assay (Dojindo, Kumamoto, Japan) according to the manufacturer’s instructions. The percentage of viable cells was calculated using the equation: cell viability (%) = (mean absorbance in test wells/mean absorbance in control wells) × 100.

### ELISA

Cell culture to measure IL-8 was carried out in the same manner as previously described (Kim et al., 2009). The indicated amounts (0, 0.5, 1, and 2 μg/ml) of cardamonin and helichrysetin were pre-incubated with dTCTP (8 μg/ml) for 15 min, then added to the cells in 1% penicillin-streptomycin/bronchial epithelial cell basal medium (BEBM), and the cultures incubated for 20 h. IL-8 secreted into the medium was measured by ELISA using a commercial kit (BioLegend).

The IL-4, IL-5, IL-13, and IFN-γ contained in the BALF were measured using specific mouse IL-4 (BioLegend), IL-5 (Thermo), IL-13 (R and D system), ELISA kits. Serum ovalbumin (OVA)-specific IgE was also measured using an ELISA kit (BioLegend). All ELISAs were performed according to the manufacturer’s instructions.

### SPR Assay

The binding of cardamonin to dTCTP was measured using a Reichert SR7500DC instrument (Reichert Technologies, Depew, NY, United States). dTCTP in 10 mM sodium acetate buffer pH 5.0 was immobilized using standard amino coupling at 15 μL/min on a 500,000 Da carboxymethyl dextran hydrogel surface sensor chip (Reichert Technologies, Depew, NY, United States). The running buffer used in all experiments was PBS pH 7.4 (2% DMSO). All SPR experiments were performed at 25°C. Cardamonin (6.25, 12.5, 25, 50, 100, and 200 μM) were injected over the dTCTP chip at 30 μL/min for 5 min. Complete dissociation of cardamonin and dTCTP was achieved after 8 min. The binding of cardamonin and dTCTP, was detected as a change in the refractive index at the surface of the chip as measured by the response units (Hakonarson et al., 1999). A reference flow cell was used to record the background response, and the background was subtracted from each of the measured RU values. The K_d_ values were calculated as the ratios of K_a_/K_d_ determined from the kinetic experiments. The data were fitted using SCRUBBER-2. This experiment was carried out by Woo Jung BSC Inc. (Suwon, Korea).

### Mouse Model of OVA-Induced Allergic Airway Inflammation

Female BALB/c mice (5–6 weeks and weighing 18–20 g) were purchased from the Central Lab. Animal Inc. (Seoul, Korea). Before experiments, all mice were allowed to become accustomed to their new environment for 1 week and were supplied with standard rodent feed and tap water. The animal room was maintained at 60–80% relative humidity at room temperature under a 12/12 h light/dark cycle. All animal studies were approved by Ewha Women’s University Institutional Animal Care and Use Committee (Approval ID: 18035).

Female BALB/c mice, 6–7 weeks of age, were injected intraperitoneally with 100 μL (0.5 mg/ml) of OVA (Thermo) conjugated with the same amount of alum (Thermo), on days 1 and 14. Two weeks after the second injection, the animals were challenged with 20 μL of saline (sham) or 100 μg of OVA, injected into the airway via their nasal cavities on days 28, 30, 32, and 34.

### BALF Collection and Inflammatory Cell Count

Mice were anesthetized, the trachea cannulated, while the thorax was gently massaged. The lungs were lavaged, three times, with 0.6 ml of phosphate buffer. The collected lavage fluid was cooled on ice and centrifuged at 1,000 × *g* for 5 min at 4°C. The supernatant was stored at −80°C for ELISA assays. The pellets were resuspended in 0.1 ml phosphate buffer, and the total inflammatory cell numbers were assessed using a hemavet (Drew Scientific Inc., Oxford, CT, United States).

### dTCTP Detection in BALF

To detect dTCTP in BALF, western blotting carried out in the same method as previously described (Kim et al., 2009). The dTCTP levels in the samples were analyzed by immunoblotting using a polyclonal rabbit anti-TCTP antibody (LabFrontier Inc., Seoul, Korea).

### Histological Analysis

Standard procedures (Lin et al., 2014) were employed for the fixation, preparation of tissue sections, deparaffination, hematoxylin, and eosin (H&E) staining, and periodic acid-Schiff staining (PAS). The densities of total inflammatory cells in the peribronchial areas of the mice from different groups were assessed using an inflammatory score from 0 to 4 on a semiquantitative scale; 0 meant no inflammation, 1 meant occasional ruffling with inflammatory cells, 2 indicated a state in which 1 to 3 layers of inflammatory cells surrounded the peri-bronchial areas, 3 meant 4 to 5 layers, and 4 meant 5 layers or more (Henderson et al., 2005; Aich et al., 2012; Hsu et al., 2012). Mucus occlusion of the airway was assessed on a scale of 0–4, where 0 indicated no mucus, 1 meant that about 10% of the bronchial diameter was blocked, 2 meant 30% occlusion, 3 meant 50% occlusion, and 4 meant greater than 80% occlusion (Henderson et al., 2005).

### Protein Extraction from the Lung Tissue and Western Blot Analysis

Protein extraction from mouse lung tissue was also performed in the same manner as in the previous experiment (Pyun et al., 2018). Proteins (10 μg) from the lung tissue were made sample for electrophoresis, separated by 10% SDS PAGE. The proteins were then transferred electrophoretically to NC membranes (GE Healthcare). The blotted membranes were blocked with 5% skim milk in TBS buffer (10 mM Tris HCl, 150 mM NaCl) at room temperature for 1 h and then probed with anti-IκB-α, anti-phospho-IκB-α, and anti-GAPDH antibodies at 4°C overnight. Antibodies were purchased from Cell Signaling Technology (Beverly, MA, United States). The blots were then washed three times with TTBS buffer (10 mM Tris HCl, 150 mM NaCl, 0.05% Tween 20) and incubated with the appropriate horseradish peroxide conjugated secondary antibodies at room temperature for 1 h. The membrane was finally washed three times, and the signals were developed using an enhanced chemiluminescent (ECL) reagent (Amersham Bioscience, Freiburg, Germany) and UV Products Imaging System, LAS 3000 (Fuji, Japan) according to the manufacturer’s instructions.

### Statistical Analysis

All results are expressed as mean ± standard error of mean (S.E.M.). Statistical analysis was performed using the Prism statistical analysis program (GraphPad 5.01). Dunnet’s test was used for the statistical comparisons of multiple groups (>*n* = 3). *p* < 0.05 was considered significant for all tests. Statistical significance is shown as *p < 0.05; **p < 0.01.

## Results and Discussion

### Cardamonin Inhibits dTCTP Induced IL-8 Release From BEAS-2B Cells

**FIGURE 1:**
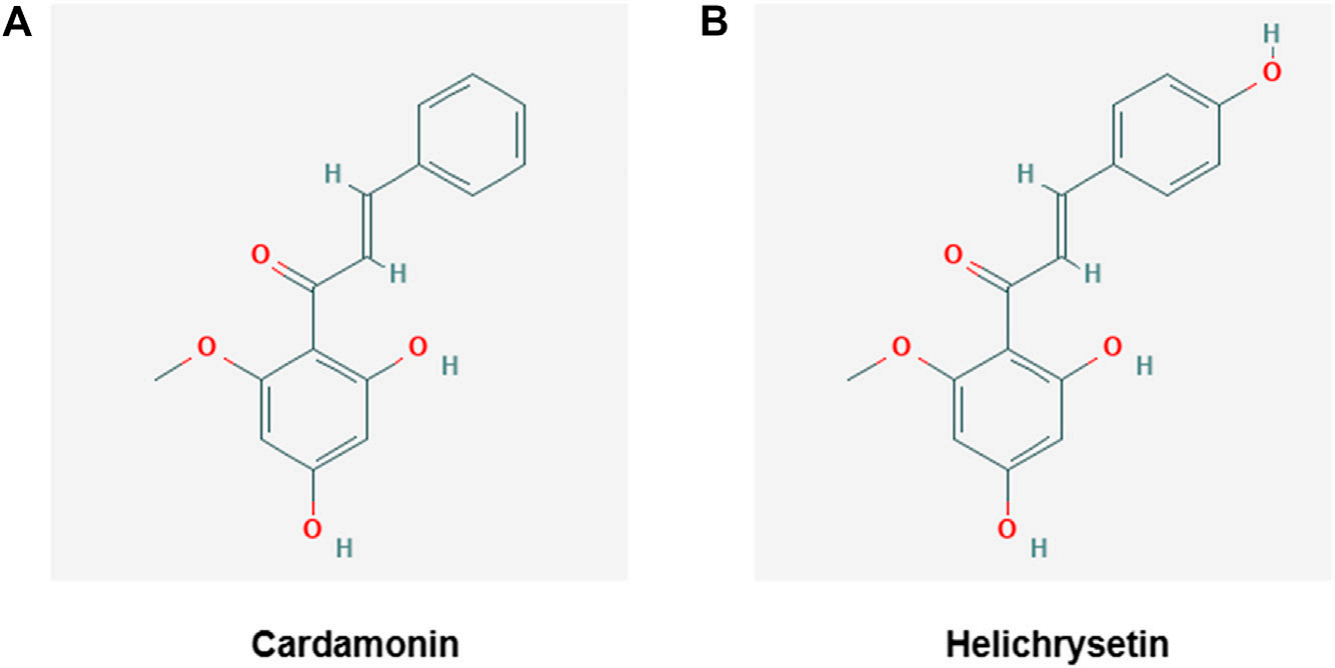
Structures of cardamonin **(A)** and helichrysetin **(B)**.

We screened a plant extract library as well as a single compound library of natural origin to identify dTCTP modulators. Through this process, cardamonin and helichrysetin wsere identified as potential dTCTP attenuators **(** Figures 1A,B **).**

**FIGURE 2:**
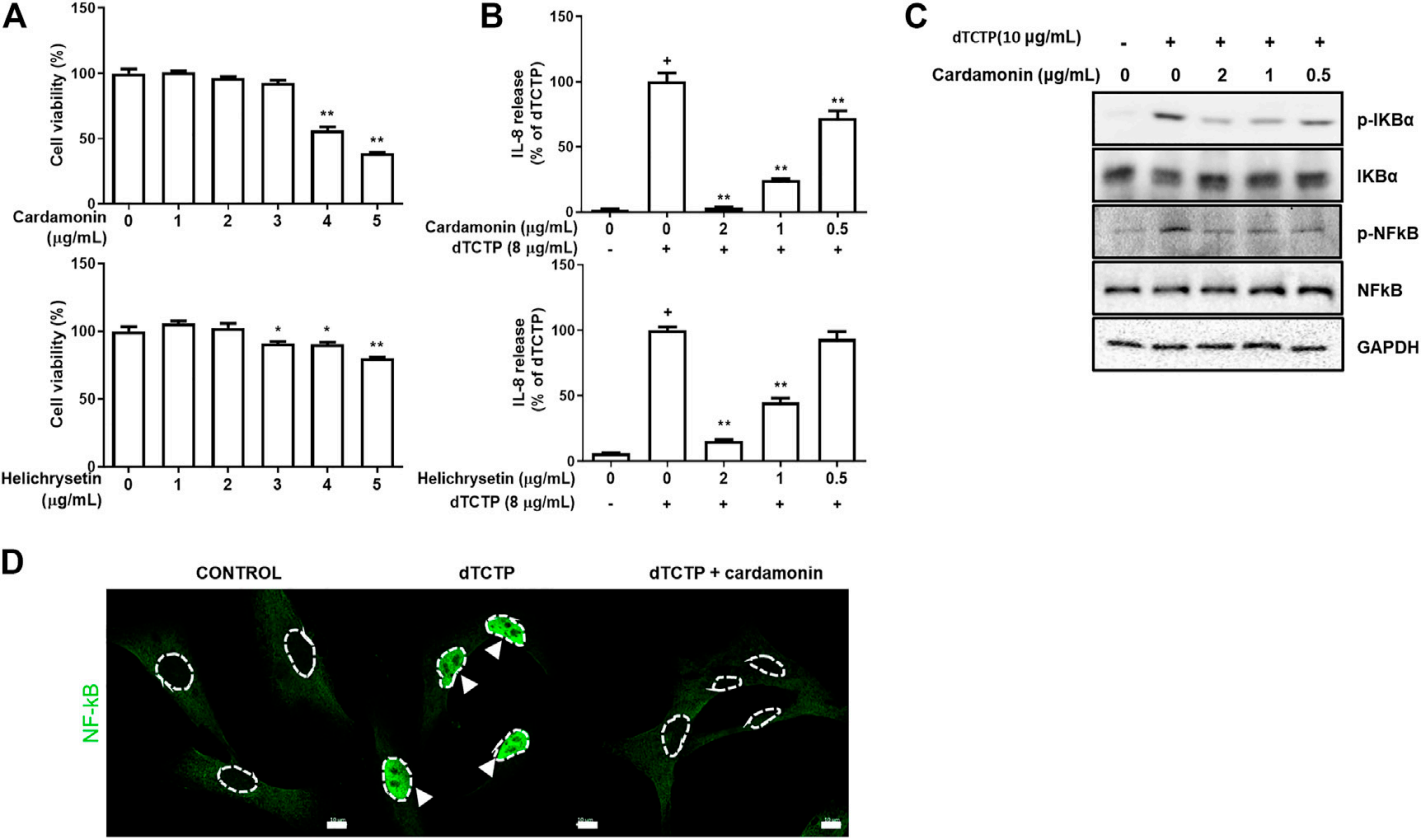
**Anti-inflammatory effects of cardamonin and helichrysetin *in vitro* studies.** Cytotoxicity of cardamonin and helichrysetin **(A)** in BEAS-2B cells. Cells were seeded into 96 well plates and treated with various concentrations of cardamonin for 24 h. Cell viability was assessed using a CCK-8 assay. The ability of cardamonin and helichrysetin **(B)** to inhibit IL-8 secretion stimulated by dTCTP in BEAS-2B cells. The effect of cardamonin on NF-κB activation during the dTCTP induced IL-8 secretion in BEAS-2B cells. Western blotting detection of family of p-IKBα, IKBα, p-NFκB, and NFκB in BEAS-2B cells. BEAS-2B cells were treated with vehicle as a negative control, dTCTP alone, or dTCTP plus cardamonin (0.5, 1, or 2 μg/ml) for 90 min **(C)**. dTCTP induced nuclear translocation of NFκB was inhibited with cardamonin (2 μg/ml) in confocal microscopy **(D)**. These values are expressed as the mean ± SEM. *n* = 3, ^+^ *p* < 0.05 compared with the negative control; *p < 0.05, **p < 0.01 compared with compared with the group treated with the dTCTP alone.

To assess the inhibitory effects of cardamonin and helichrysetin on the dTCTP-induced secretion of IL-8 in BEAS-2B cells, we tested their dose-dependent effects on BEAS-2B cell viability **(** Figure 2A). Neither substance showed toxicity at concentrations below 2 μg/ml. Next, we examined their effect on IL-8 secretion caused by dTCTP in BEAS-2B cells using a concentration range, where no cytotoxicity was observed. Both of them decreased the dTCTP-induced secretion of IL-8 in BEAS-2B cells in a dose-dependent manner (Figure 2B).

Based on the results from Figure 2, we performed *in vivo* tests using cardamonin or helichrysetin. The tests showed that helichrysetin was not effective in our allergic airway inflammation mouse model. Although booth cardamonin and helichrysetin have the similar structures, helichrysetin has one more OH group in the ring. Therefore, the aromatic ring without OH group seems to be necessary for *in vivo* activity. Thus, helichrysetin was excluded from further *in vivo* studies and we focused on cardamonin.

### Cardamonin Inhibits the dTCTP-Induced Activation of the NF-κB Signaling Pathway in BEAS-2B Cells

Previous studies have shown that dTCTP causes the inflammatory response by activating the NF-κB pathway (Lee and Lee, 2018) and that cardamonin modulates inflammation by inhibiting NF-κB activity (Li et al., 2015). Based on previous results, we performed western blotting assay to confirm changes in NF-κB activity and found that the phosphorylation of IκB-α was prevented in BEAS-2B cells treated with cardamonin in a dose-dependent manner, and statistical significance was confirmed in the group treated with 2 μg/ml cardamonin (Figure 2C). In agreement with the immunoblotting results, the effect of cardamonin treatment on the dTCTP–stimulated nuclear translocation of NF-κB in BEAS-2B cells was examined by immunofluorescence analysis with antibody to NF-κB. For untreated cells stimulated either with dTCTP or cardamonin, most immune reactivity for anti-NF-κB was detected in the cytosol, with only a small amount apparent in the nucleus. Stimulation with dTCTP resulted in a marked decrease in anti-NF-κB immunoreactivity in the nucleus in cardamonin treated cells but not in untreated cells. These results thus suggest that cardamonin is an effective inhibitor of the nuclear translocation of NF-κB (Figure 2D).

### Cardamonin Binds to dTCTP

Since cardamonin has been shown to inhibit the effects of dTCTP on IL-8 secretion, we hypothesized that cardamonin directly or indirectly influences the role of dTCTP. To confirm that two substances bind, we conducted kinetic analysis of the interaction between dTCTP and cardamonin using surface plasmon resonance spectrometry (SPR).

The equilibrium dissociation constant (K_D_) of cardamonin for dTCTP was 4.72 ± 0.07 μM **(** Table 1 **).** This confirmed binding of cardamonin to dTCTP and that this binding seems to inhibit the histamine releasing activity of dTCTP.

**TABLE 1:** Kinetic binding parameters for cardamonin. Cardamonin binding to dTCTP determined by SPR (Reichert SPR SR7500 instrument).

Substance	Ligand	Kinetic binding parameters		
		K_a_ (M^−1^s^−1^)	K_d_ (s^−1^)	K_D_ (μM)
Cardamonin	dTCTP	110 ± 0.2	5.2e^−4^ ± 0.01e^−4^	4.72 ± 0.07

Numbers in parentheses represent the standard error of the kinetic fit.

Numerous studies have shown that TCTP binds to a number of pharmacologically active substances. For example, there is evidence that TCTP binds to antihistamine agents, such as levomepromazine (57.2 ± 6.49 μM) and buclizine (433 ± 47.1 μM) (Tuynder et al., 2004; Seo and Efferth, 2016). The dehydrocostus lactone used in our previous study is known to interact with dTCTP (5.33 ± 0.03 μM) (Pyun et al., 2018). Based on these results, we assumed that cardamonin plays the role as antihistamine agents by binding to dTCTP.

### Cardamonin Reduces Inflammatory Cells in BALF

We used a mouse allergic airway inflammation induced by OVA to determine the efficacy of cardamonin in the treatment of allergic airway inflammation. OVA is commonly used as an antigen for generating mouse models of allergic airway inflammation (Fuchs and Braun, 2008; Yao et al., 2016; Zhang et al., 2016; E.; Zhou et al., 2014). OVA reliably induces TH_2_ allergic responses (Zosky and Sly, 2007). To confirm the anti-inflammatory effect of cardamonin, mouse BALF were first evaluated for the presence of inflammatory cells.

**FIGURE 3:**
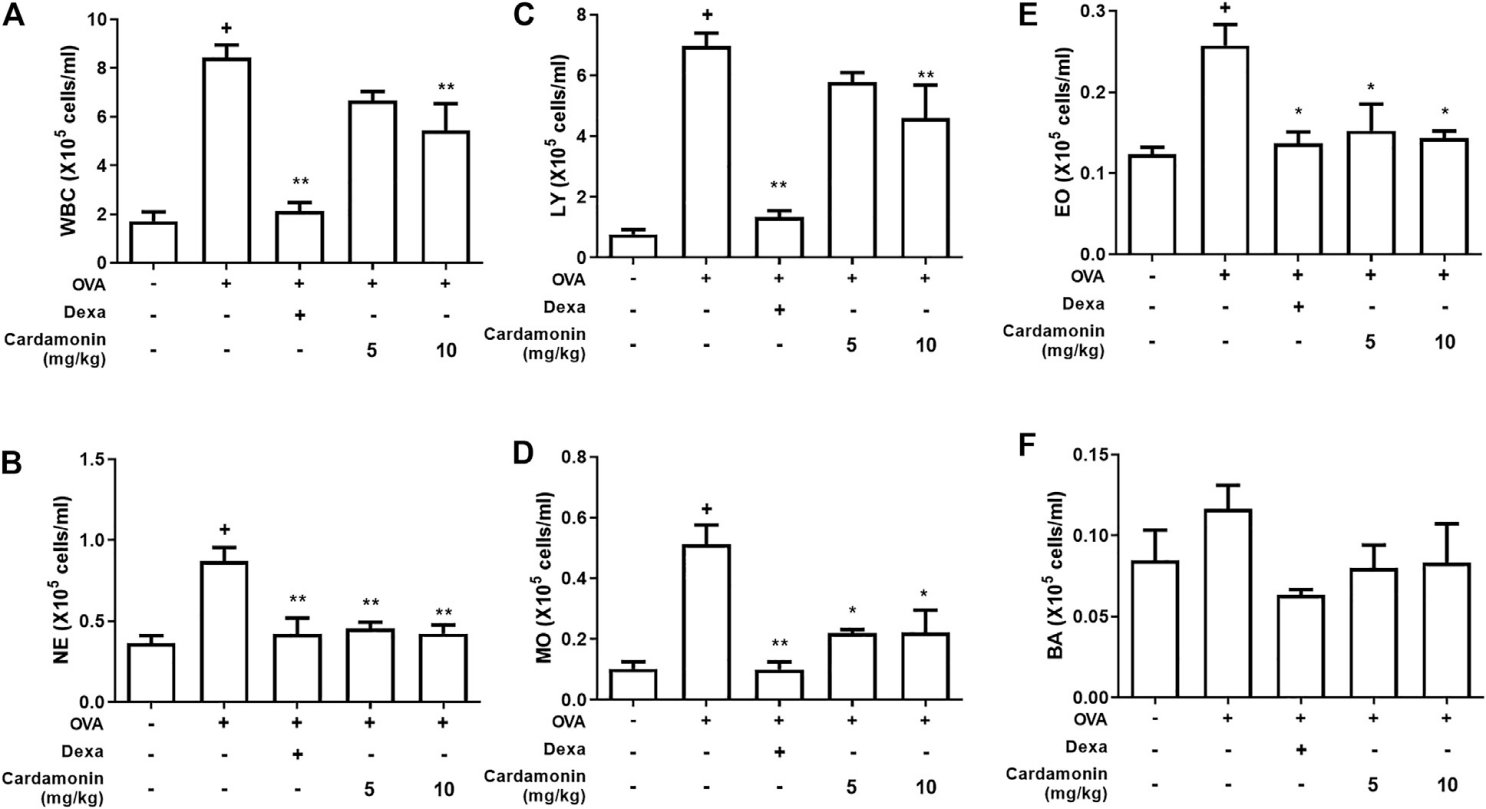
**Cardamonin decreases the recruitment of inflammatory cells in BALF.** BALB/c mice were treated with cardamonin (5 or 10 mg/kg) or vehicle (2% DMSO in saline) daily from 28th to the 34th day after the first immunization with OVA. Dexamethasone (1 mg/kg) was used as positive control. WBC, white blood cells; NE, neutrophils; LY, lymphocytes; MO, monocytes; EO, eosinophils; BA, basophils. The values presented are means ± SEM. *n* = 3–6, ^+^ *p* < 0.05 compared with the sham group; *p < 0.05, **p < 0.01 compared the vehicle group treated with OVA.

For this, we employed four groups of mice. Two groups received 5 mg/kg and 10 mg/kg cardamonin dissolved in vehicle, respectively; the third group received only the vehicle and the fourth received 1 mg/kg dexamethasone for a positive control. Dexamethasone is a glucocorticoid that inhibits the expression of inflammatory genes, such as nuclear factor-κB (NF-κB), which are activated during inflammatory response and increase the expression of cytokines, chemokines, and adhesion molecules (Barnes, 2006). In the vehicle group, various inflammatory cells were found to be increased, confirming that OVA treatment caused inflammatory response in the airway. Both the groups that received cardamonin by intraperitoneal injection showed significantly reduced numbers of eosinophils, neutrophils, monocytes, and basophils at 5 mg/kg and 10 mg/kg to a similar extent (Figures 3B, D−F). But 10 mg/kg injection of cardamonin inhibited white blood cells and lymphocytes than 5 mg/kg injection (Figures 3A,C).

### Cardamonin Regulates the Level of Inflammatory Cytokines in BALF

We examined how cytokines in BALF changed in the mouse allergic airway inflammation model. For this, the levels of TH_2_ cytokines, IL-4, IL-5, and IL-13, typically seen in allergic inflammation, were determined by ELISA. TH_2_ cytokines are key players in allergic disorders.

**FIGURE 4:**
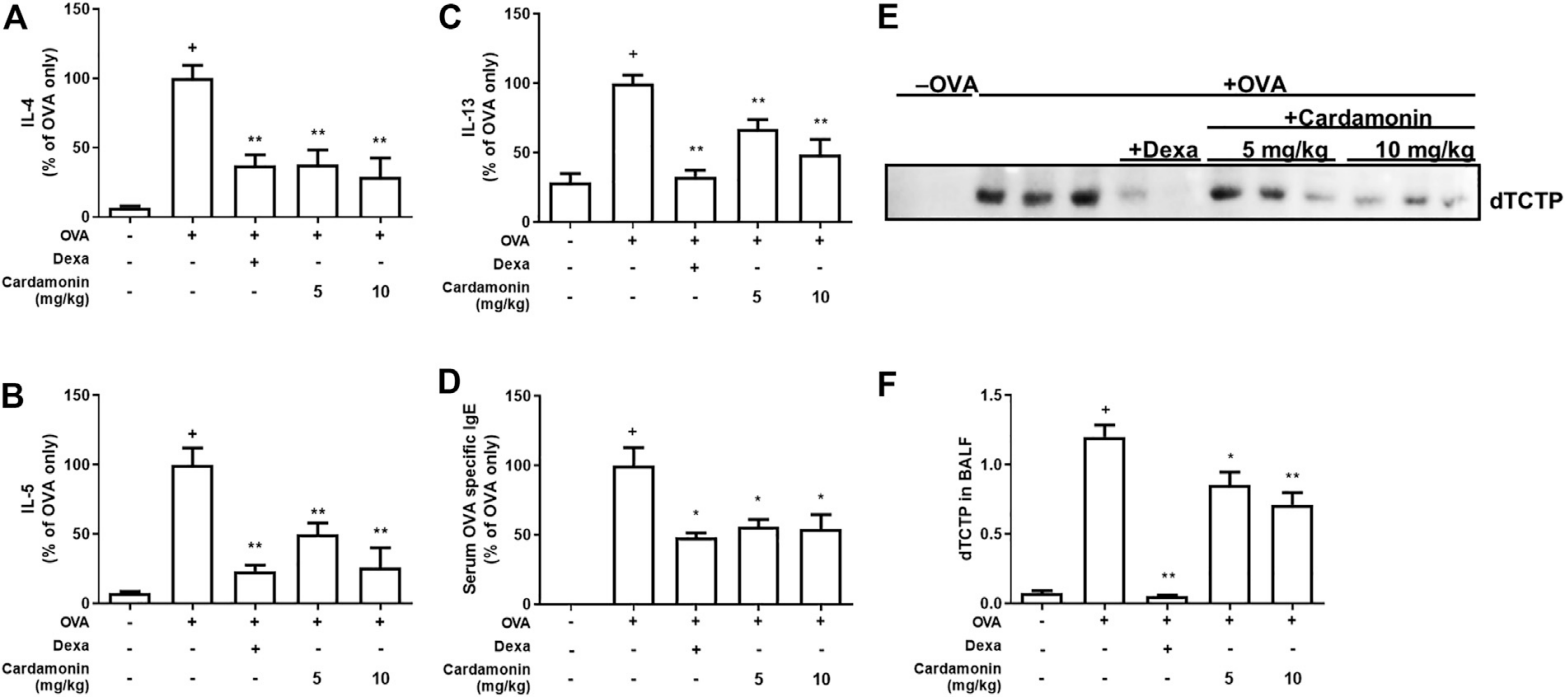
**Cardamonin has anti-inflammatory effects in a dose dependant manner.** The levels of IL-4 **(A)**, IL-5 **(B)**, IL-13 **(C)**, and serum levels of OVA-specific IgE **(D)** were measured by ELISA after sacrifice. The levels of IL-4, IL-5, IL-13, and OVA specific IgE were increased significantly in OVA-treated mice; however, the increased levels of these cytokines were significantly suppressed by cardamonin treatment. BALF was concentrated and immunoblotted for TCTP **(E)**. The quantitative results are shown in **(F)**. The values shown are means ± SEM. *n* = 3–6, ^+^ *p* < 0.05 compared with the sham group; *p < 0.05, **p < 0.01 compared with the vehicle group treated with OVA.

The levels of IL-4, IL-5, and IL-13 were elevated in the allergic airway inflammatory group. However, the levels of these cytokines were significantly decreased in groups receiving 5 or 10 mg/kg cardamonin. Overall, cardamonin down-regulated these OVA-induced TH_2_ cytokines (IL-4, IL-5, and IL-13) in a dose-dependent manner (Figures 4A–C).

### Cardamonin Reduces OVA-Specific Serum IgE Levels

If cardamonin plays a role in controlling allergic airway inflammation, the levels of IgE which are crucial for the development of allergic responses will also decrease. Serum IgE levels were elevated in all groups except the sham group. Among the OVA-induced allergic airway inflammation groups, serum OVA-specific IgE levels were significantly reduced by more than 40% in mice treated with 5 or 10 mg/kg cardamonin compared to mice treated with vehicle alone (Figure 4D), suggesting that cardamonin reduces the IgE-mediated allergic response.

### Cardamonin Regulates Secretion of dTCTP in BALF

We examined the changes in dTCTP levels in BALF from mice treated with cardamonin. All mice in various groups, except the sham group, contained dTCTP in their BALF, consistent with our previous observations (Kim et al., 2009), confirming that cardamonin reduces dTCTP. In OVA-induced allergic airway inflammation groups, dTCTP was significantly reduced in mice treated with 5 or 10 mg/kg cardamonin compared to mice treated with vehicle alone (Figures 4E,F). This may be because cardamonin improves the allergic environment that suppresses dimerization of TCTP and cytokine release from basophilic cells. Another possibility is cardamonin reduces dTCTP by decreasing the exocytosis of TCTP from cells like macrophages or by transcriptionally or translationally regulating intracellular levels of TCTP.

### Cardamonin Ameliorates Histopathological Changes in Lung Tissue

Allergens cause pathophysiologic changes in vascular permeability, bronchoconstriction, epithelial hypertrophy, and mucus production (Galli et al., 2008; Holgate, 2008). Hypertrophy and hyperplasia of smooth muscle cells of the bronchus are common phenotypes in allergic airway diseases. These changes in smooth muscle cells cause airway hyper-responsiveness, one of the hallmarks of asthma. In particular, goblet cell hyperplasia stimulates the secretion of mucus into the airway lumen and promotes airway remodeling (Aikawa et al., 1992; Rogers, 2007).

**FIGURE 5:**
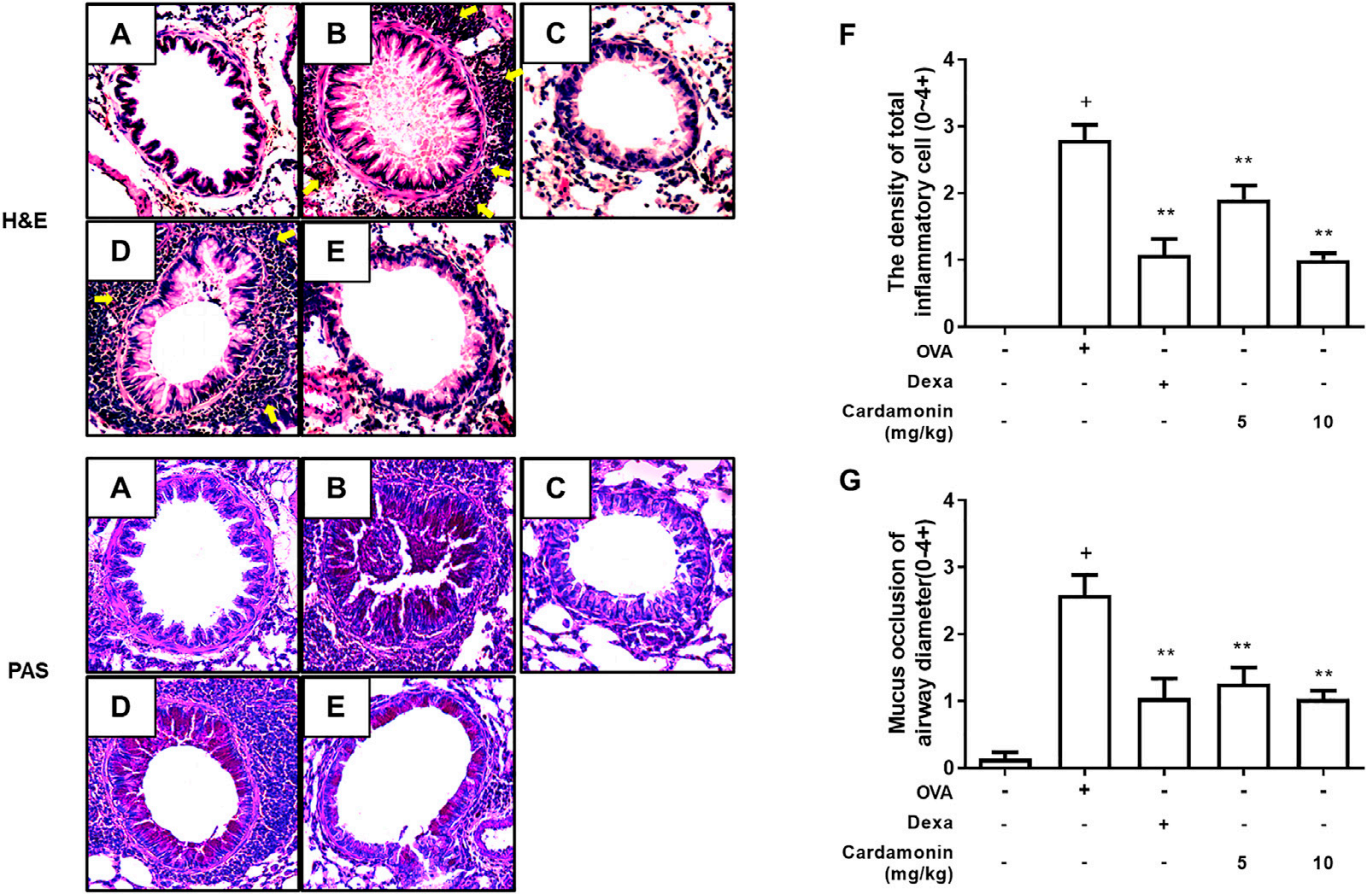
**The effect of cardamonin on histopathological changes in the lung tissues of OVA-induced allergic airway inflammation model.** Inflammation was evaluated in the lung tissues by analyzing inflammatory cell infiltration (H&E staining) and goblet cell hyperplasia (PAS staining). Representative lung tissue sections from the sham group **(A)**, the OVA sensitized and challenged mice treated with vehicle **(B)**, dexamethasone (1 mg/kg) **(C)**, or with cardamonin (5 and 10 mg/kg) **(D and E)**. The number of peri-bronchial inflammatory cell was upregulated in mice receiving the vehicle compared with mice receiving sham treatment **(F)**. Mucus occlusion was significantly attenuated in mice of allergic airway inflammation treated with cardamonin **(G)**. The H&E and PAS stained sections are magnified ×200. The values presented are means ± SEM. *n* = 3–6, ^+^ *p* < 0.05 compared with the sham group; *p < 0.05, **p < 0.01 compared with the vehicle group treated with OVA.

To characterize the effects of cardamonin on lung tissue in the OVA-induced mouse allergic airway inflammation model, lung sections were stained with Hematoxylin and Eosin (H&E) and Periodic Acid-Schiff (PAS). Treatment with cardamonin significantly inhibited inflammatory cell influx, indicating that cardamonin is effective in attenuating the pathophysiological processes in allergic airway inflammation (Figure 5). The OVA-stimulated mice showed a significant increase in the number of mucus and goblet cells in the bronchial airways compared with the sham group. In contrast, the number of mucus and goblet cells was significantly reduced in a dose-dependent manner in the cardamonin treated groups (Figure 5).

### Cardamonin Inhibits the Phosphorylation of IkB

**FIGURE 6:**
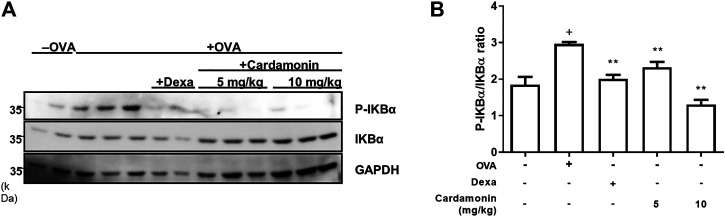
**Effect of cardamonin on phosphorylation of IkBα in OVA-induced allergic airway inflammation model in BALB/c mice**. Western blotting was used to detect phosphorylated and total IkB-α levels in lung tissue from mice in the sham group, and the OVA-sensitized and OVA re-challenged mice treated with vehicle, dexamethasone (1 mg/kg), or cardamonin (5 and 10 mg/kg) **(A)**. The quantitative results are shown in **(B)**. The values presented are means ± SEM. *n* = 3–6; ^+^ *p* < 0.05 compared with the sham group; *p < 0.05, **p < 0.01 compared with the vehicle group treated with OVA.

It is well-known that cardamonin is involved in inflammatory signaling pathways, such as the NF-κB and MAPK pathways (Shen et al., 2014; Li et al., 2015). In addition, dTCTP promotes IL-8 secretion in BEAS-2B cells by activation of the NF-κB pathway (Lee and Lee, 2018). We hypothesized that cardamonin would inhibit the activation of NF-κB caused by dTCTP. We performed western blotting to measure changes in NF-κB activity and found that cardamonin inhibits the activation of NF-κB by suppressing the phosphorylation of IκB-α in lung tissues of OVA-induced allergic airway inflammation mouse model (Figure 6). Phosphorylation and degradation of IκB-α were effectively prevented in mice treated with cardamonin.

Thus, we confirmed that cardamonin is effective in treating allergic lung inflammation in mice. Many other natural products have been shown to inhibit airway inflammation and many others are under development. For example, DW 2008S (Youm et al., 2018), a new natural product identified by Donghwa Pharmaceuticals is in Phase I clinical trial. DW 2008S is extracted from *Justicia procumbens* and has a bronchoconstriction effect and inflammation control. Other agents that have recently been evaluated in clinical trials include an antibody directed against thymic stromal lymphopoietin (Tezepelumab) (Ying et al., 2005), small molecule antagonists of the chemo attractant receptor-homologous molecule expressed on TH2 cells (Fevipiprant) (Gonem et al., 2016) and the receptor for stem cell factor on mast cells (Imatinib) (Cahill et al., 2017), and a DNA enzyme directed at GATA3 (Krug et al., 2015). Antibodies to IL-33 and its receptor, ST2, are also being evaluated in ongoing clinical studies (Queiroz et al., 2017). In addition, many other potential candidates are being considered for future clinical trials.

## Conclusion

Dimerized TCTP (dTCTP) plays an important role in the allergic inflammation by promoting the secretion of histamine and cytokines in the late phase reaction. Substances that inhibit these secretions by interacting with dTCTP can be one such in the therapy of allergic diseases. This study demonstrated that cardamonin, a chalcone identified from a library containing various chemicals from natural plants, inhibits allergic reactions caused by dTCTP. Cardamonin inhibited dTCTP-induced IL-8 secretion in human bronchial cell lines, and SPR confirmed that cardamonin interacts with dTCTP. In the mouse allergic airway inflammation model, cardamonin reduced inflammatory cells and the secretion of dTCTP in BALF, an indicator of improvement of the inflammatory response. These results suggest that cardamonin can be considered as a potential candidate drug for alleviating allergic inflammation.

## Data Availability

The original contributions presented in the study are included in the article/Supplementary Materials, further inquiries can be directed to the corresponding authors.

## Abbreviations

BALF, Bronchoalveolar lavage fluid; dTCTP, Dimerized translationally controlled tumor protein; ELISA, Enzyme-linked immunosorbent assay; HRF, Histamine releasing factor; NF-κB, Nuclear factor kappa B; OVA, Ovalbumin; SPR, Surface plasmon resonance.
